# Traversing the Valley of Death for nanotechnology-based natural products: strategies and insights from pharmaceutical stakeholders

**DOI:** 10.1007/s13346-025-01923-8

**Published:** 2025-07-18

**Authors:** Jian Sheng Loh, Chan Yew Low, Wei Peng Low, Yun Lin Ng, Wen Qi Mak, Li Kar Stella Tan, Hong Hao Chan, Yong Sze Ong, Stephanie Kay Ann Cheah, Emmellie Laura Albert, Chee Wun How

**Affiliations:** 1 School of Pharmacy, Monash University Malaysia, Jalan Lagoon, Selatan Bandar Sunway, Selangor, 47500 Malaysia; 2 School of Pharmacy, Faculty of Health & Medical Sciences, Taylor’s University, 1, Jalan Taylors, Subang Jaya, Selangor 47500 Malaysia; 3 Digital Health and Medical Advancements Impact Lab, Taylor’s University, Subang Jaya, Selangor 47500 Malaysia; 4 School of Business and Economics, Monash University Malaysia, Jalan Lagoon Selatan, Bandar Sunway, Selangor 47500 Malaysia; 5 Institute of Nanosciences and Nanotechnology, Universiti Putra Malaysia, Serdang, Selangor 43400 Malaysia

**Keywords:** Nanotechnology, Natural products, Pharmaceutical innovation, Commercialisation, Valley of Death, Translational framework

## Abstract

Nanotechnology-based natural products hold significant therapeutic promise by improving the bioavailability and efficacy of natural products. However, their translation from laboratory to clinic remains hindered by a complex interplay of strategic misalignment, regulatory complexity, and organisational hurdles, often leading to failure in the so-called “Valley of Death”. To understand the drivers of successful translation and commercialisation, this study engaged 16 pharmaceutical stakeholders through semi-structured, in-depth interviews. Thematic analysis revealed three critical enablers. First, product-market fit requires early integration of market analysis and need-driven innovation to ensure alignment with unmet medical needs and commercial viability. Moreover, effective articulation of value propositions is essential to translate technical performance into perceived value for both investors and end-users. Second, product differentiation and positioning are essential to communicate competitive advantage in a crowded and cost-sensitive market. Strategic use of comparators, value metrics, and strategic messaging are critical to demonstrate tangible benefits over existing formulations, while avoiding unnecessary complexity to preserve affordability. Third, cross-sector and horizontal collaborative networks across academia, industry, regulators, manufacturers, and peer companies play a vital role in overcoming translational challenges by fostering regulatory preparedness and pooling complementary expertise and resources. Building on these findings, we propose the NATURAL framework as a translational planning tool to support early decision-making by helping innovators anticipate key barriers, align strategies, and improve the likelihood of clinical translation. Altogether, this study offers actionable insights and a structured planning tool to de-risk and accelerate the translation of nano-based natural products from bench to market.

**Graphical Abstract:**

**Figure:**
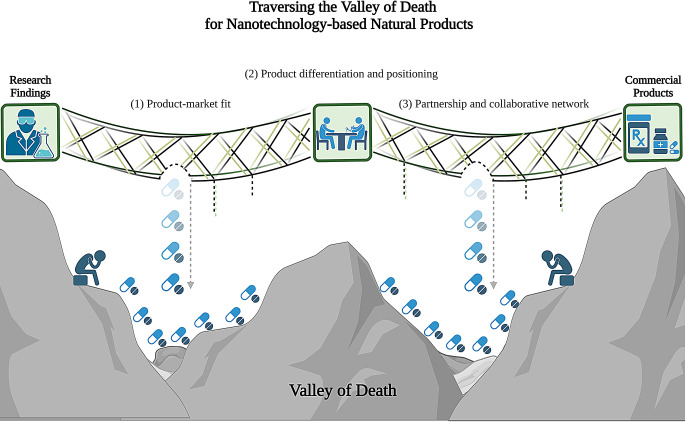

## Introduction

Nanotechnology has emerged as a transformative tool in the biomedical and pharmaceutical sciences, owing to its unique physiochemical and structural characteristics that address many of the limitations associated with conventional drug delivery systems [1, 2]. It involves engineering materials at the nanoscale (1–100 nm), enabling a vast array of applications due to the emergence of unique physical, chemical, and biological properties that differ from those of their bulk counterparts [3]. As of 2024, a total of 5,367 nano-based products were registered in the European market, a nearly fourfold increase from 2012, with 3,272 (60.96%) classified under the ‘Health and Fitness’ category [4]. These trends underscore the growing integration of nanotechnology into consumer and healthcare products, particularly in formulations designed to enhance human health.

Natural products have historically served as a prominent source of drugs, largely due to their exceptional scaffold diversity and structural complexity [5]. However, major pharmaceutical companies have shifted away from natural product-based drug discovery due to several drawbacks, including high cost, difficulties in sourcing sufficient quantity, laborious isolation and purification processes, challenges in scalability, and concerns regarding novelty and intellectual property (IP) [5, 6]. Moreover, many natural products suffer from poor aqueous solubility, chemical instability, low permeability across biological barriers, non-specific targeting, and low bioavailability, all of which contribute to limited clinical efficacy [7, 8]. Nevertheless, the past two decades have witnessed a remarkable resurgence of interest in natural products, driven by the vast therapeutic potential offered by their structural complexity, enhanced by recent technological advances, particularly in nanotechnology [5, 6].

Nanotechnology offers significant promise for advancing the clinical translation of natural products, owing to its ability to protect bioactive compounds from degradation, enhance solubility, enable targeted delivery, control drug release, and improve therapeutic efficacy and safety [7, 8]. Despite these potentials, the transition from innovation to commercial product has been limited. This has deepened concerns about the real-world value of nano-based formulations and severely discouraging prospective investors from funding nanotechnology projects. A notable exception is the rapid development of the Pfizer–BioNTech and Moderna COVID-19 mRNA vaccines, which entered clinical trials merely 3 months after acquiring the SARS-CoV-2 genome sequences and were subsequently utilized at the global level [9]. This success over traditional and established vaccine technology has validated the potential of nanotechnology in medicine, which has improved the confidence of various stakeholders in accepting and adopting nanotechnology in medicine.

Despite intensive research efforts and promising preclinical results, the number of nano-based natural products that have achieved commercial success and real-world impact remains dismally low. The persistent gap between innovation and successful commercial products underscores the limitations of focusing solely on scientific excellence. Notably, nearly a quarter of the investigational drug failures were attributed to commercial and strategic reasons [10, 11]. These statistics highlight the need for insights from experienced pharmaceutical stakeholders who understand the practical hurdles that must be overcome for successful translation of nano-based natural products to the market.

To address this critical gap, this study investigates the commercialisation challenges of nano-based natural products based on insights from pharmaceutical stakeholders, thereby offering strategic insights to guide nano-based natural products across the Valley of Death. The central research question guiding this study was: What strategies enable the successful commercialisation of nano-based natural products? To answer this, we conducted a qualitative study exploring the perspectives of stakeholders from pharmaceutical industries and pharmacies, using a series of elicitation questions to capture their insights.

## Methods

### Study design and recruitment

This study adopted a qualitative research design to explore the perspectives of stakeholders involved in the development and commercialisation of nano-based natural products. Semi-structured, in-depth interviews were conducted to allow participants the flexibility to express their views within the boundaries of the research topic. Purposive sampling was applied to acquire maximum variation in stakeholder perspectives and experiences [12]. Recruitment continued until thematic saturation was reached. It is defined as the point at which no new or relevant insights emerged that would further inform the research question [13].

Purposive sampling was used to recruit stakeholders across diverse sectors, including manufacturing and non-manufacturing pharmaceutical companies, biopharmaceutical and nutraceutical industries, retail pharmacy, and digital health. Participants held roles spanning marketing, business development, research and development, clinical research, and community pharmacy practice, with several occupying senior leadership positions. Eligible participants were identified through professional networks such as LinkedIn and invited based on their relevant expertise or experience with product development and commercialization. Publicly available professional profiles were reviewed to assess eligibility, and additional background information was obtained from participants to confirm their suitability. A total of 16 respondents were recruited to participate in the interviews. The inclusion criteria were as follows:

1. Currently or previously held a position in a pharmaceutical-related company or local pharmacy registered with Pharmacy Board Malaysia.
2. Possess relevant experience in at least one of the following areas: nanotechnology, natural products, pharmaceutical research and development (R&D), formulation and production, regulatory affairs, or sales and marketing.

An explanatory statement was given to the potential respondents to ensure that they clearly understood the research objectives, the process to be followed, and measures taken to maintain their confidentiality and anonymity before agreeing to join. Written consents were obtained from the respondents prior to the interview. All study procedures were approved by the university’s ethics committee (Reference Project ID: 27759).

### Data collection

Data collection was carried out between March 2021 and June 2021. Interviews were conducted individually via video conferencing software and recorded to facilitate both the data collection and analysis. Open-ended questions were used to encourage the respondents to share their thoughts and experiences regarding the research topics. The video and audio recordings were transcribed verbatim and reviewed for accuracy.

### Data analysis

**Fig. 1:**
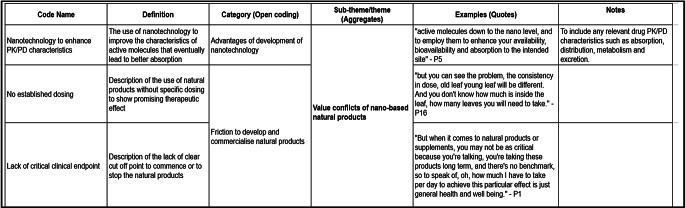
Overview of coding framework for data analysis

The data analysis occurred in tandem with data collection. Data analysis for this study was conducted collaboratively by five researchers (JSL, CYL, WPL, ELA, and YLN) under the supervision of three supervisors (CWH, YSO, and SKAC). After familiarising themselves with the transcripts, the researchers had come to consensus and developed data analysing protocol (Appendix A) as a guide for the data-analysing process. The master codebook served as a primary reference document. The researchers created the codebook in Google Sheets and included the following fields: Code name, Definition, Category (open coding), Sub-theme/Theme (aggregates), Examples (quotes), and Notes. These codes were then categorised accordingly under the corresponding key themes that structure the research findings as shown in Fig. 1.

## Results

**Fig. 2:**
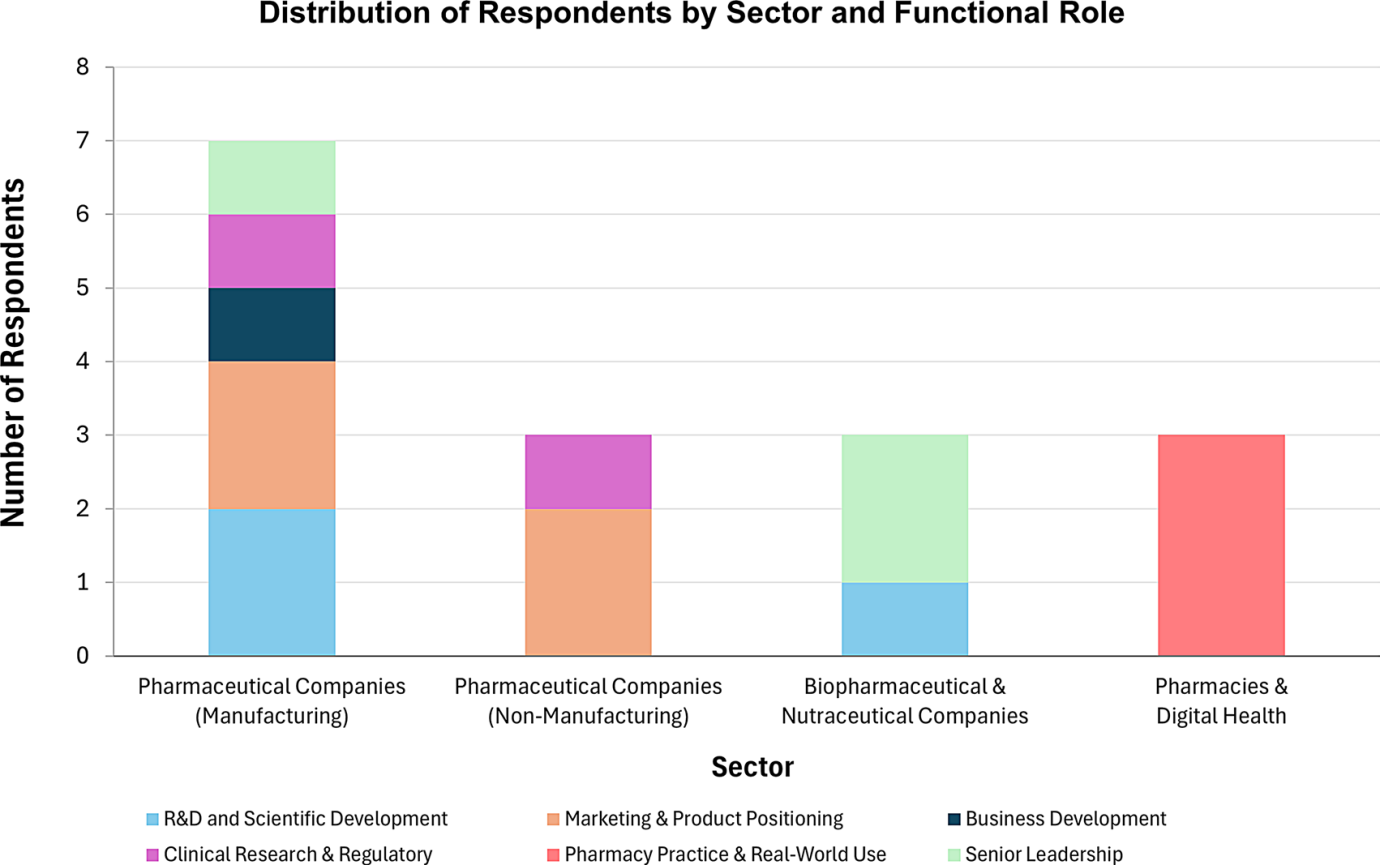
Distribution of study participants by sector and functional expertise

This study included 16 respondents, with a mean working experience of 9 years (σ = 6.71). Their professional affiliations are displayed in Fig. 2. The sample represented a wide range of functional expertise, including marketing, business development, R&D, clinical research, and community pharmacy practice, with three participants also holding senior leadership positions. It also spanned diverse sectors such as pharmaceutical manufacturing, non-manufacturing pharmaceutical companies, biopharmaceuticals, nutraceuticals, retail pharmacy, and digital health. This cross-disciplinary and cross-sectoral diversity enabled the collection of multifaceted insights on the integration of nanotechnology into natural products, encompassing both strategic perspectives (e.g. decision-making, product positioning) and operational insights (e.g. development feasibility, market fit, real-world applicability).

To protect anonymity, participants were identified using respondent codes (e.g. R1, R2, R3,…). During data analysis, sub-themes were organized under three key themes; (1) Product-market fit, (2) Product differentiation and positioning, and (3) Partnership and collaborative network.

### Key theme 1: product-market-fit

The first theme underscores the importance of conducting in-depth market analysis to assess the marketability of nano-based natural products (Table 1). Respondents highlighted that despite scientific enthusiasm, a realistic assessment of market potential is critical, as pharmaceutical R&D and nanoformulation development are costly, risky, and time-consuming. By adopting a market-aware approach to R&D planning, companies can allocate resources more strategically, reduce development risks, and remain innovative in a cost-effective manner.

A key concern raised by respondents was the lack of early market evaluation in academic-led research on nano-based natural products. Many research teams fail to assess whether their innovations address real-world clinical gaps or align with market needs, leading to products that are scientifically promising but commercially unviable. Specifically, respondents highlighted the importance of estimating market demand and size, identifying the target patient population, and developing a viable pricing strategy from the outset. One way to assess market demand is by targeting disease areas with high unmet therapeutic needs, particularly where conventional natural products have shown anecdotal and preliminary clinical promise but remain underutilized due to formulation challenges. This process involves gathering physicians’ and patients’ experiences with existing natural products, including their effectiveness in controlling disease symptoms and severity, onset of action, side effect profile, and the convenience of dosing schedule and route of administration. Unlike conventional synthetic drugs, many natural products suffer from poor solubility, low bioavailability, and inconsistent therapeutic effects, properties that have historically hindered their clinical application. The integration of nanotechnology into natural products may solve these limitations and unlock previously inaccessible market segments, such as neurodegenerative or inflammatory diseases, where blood-brain barrier penetration or localized delivery is critical. In addition, respondents emphasized the need to consider whether the enhanced performance of nano-based formulations justifies a higher price point.

Another persistent challenge identified by respondents was the disconnect between early-stage R&D efforts and commercial reality in the development of nano-based natural products. Although R&D scientists may have strong confidence and optimism in the therapeutic potential, respondents agreed that this enthusiasm must be tempered by the actual marketability and practicality. To address this, respondents emphasized the importance of integrating market research into the initial phases of R&D to inform product positioning and business planning, which serves as a strong signal to attract investors and support investment decisions. Specifically, the anticipated label claims derived from nanoformulation advantages, such as faster onset, enhanced efficacy, improved tolerability, or once-daily dosing, must be commercially compelling to justify higher pricing and appeal to potential investors. However, historical concerns over the variability of natural product efficacy and sourcing, lack of standardization, weak IP protection, and uncertain cost-effectiveness continue to make investors cautious. To overcome this, researchers must demonstrate consistent sourcing, batch-to-batch reproducibility, robust and reproducible evidence of therapeutic benefits, clear IP advantage, and evidence of cost-effectiveness through pharmacoeconomic modeling. Respondents also emphasized the need to assess whether the target audience is sufficiently large and motivated to sustain commercial returns, as this directly influences investor confidence and willingness to fund development. However, respondents noted that academic researchers often lack the market analysis expertise and understanding of investor expectations needed to articulate a compelling value proposition. Thus, it is essential for the R&D team to have regular interactions and discussions with the marketing and business development teams to review market opportunities and assess commercial viability before committing fully to a research idea. Ultimately, nanoformulation should not be pursued for novelty alone, but it must align with market realities and enable clinical translation of otherwise unusable natural products.

In summary, this theme highlights the importance of carefully incorporating market-driven insights and patient-centric design metrics to inform the research effort at every stage. Research must be guided not only by scientific potential but also by clearly articulated market needs, consumer value, and strategic planning. This enables firms to make well-informed plans of action to maximise the chances of commercial success and deliver the benefits of nano-based natural products to the consumers.

**Table 1:** Product-Market-Fit

Key Themes	Sub-Themes	Exemplar Quotations
Product-Market-Fit	Identifying unmet medical need	“We usually develop products based on what the market wants, so you don’t try and develop a product and expect the market to want it. It should be the other way around. If you want to do some new, novel research, maybe the guiding principle should be to address an unmet medical need.” (R9)
	Conducting comprehensive market analysis	“Who is our target audience, how should we market the products, how should we price the products.” (R5)
	Facilitating cross-functional collaboration between R&D and marketing	“A lot of the time, things are being done in silos. The R&D team is doing something that they think that it is good for the population, but it may be out of date. The communication between the R&D team and marketing team has to be faster” (R5)
	Developing a strategic plan to attract investors	“What is the return, what is the benefit and what is the advantage in the market, these are going to help you in generating the momentum and willingness for people to invest in this and then push forward. To get a deal done, it normally has to be of interest to both parties.” (R1)

### Key theme 2: product differentiation and positioning

In the second theme, respondents emphasized that clear and meaningful differentiation from conventional natural products is a critical determinant of commercial success (Table 2). A major challenge raised by respondents is the insufficient distinction between nano-based and conventional natural products. The market is saturated with conventional formulations, and consumers have become increasingly informed and sensitive to the features and prices of products on the market due to the rapid advancements in information technology. Without a clear understanding of existing competitors and their offerings, nano-based natural products risk failing to stand out in a crowded market.

To overcome this, respondents stressed the importance of conducting in-depth competitor landscape analyses to identify market gaps and define the therapeutic or performance limitations that can be addressed by the new nanoformulation. Tools such as SWOT (strengths, weaknesses, opportunities, and threats) analysis were recommended to assess competitors’ product attributes, pricing, target demographics, and market positioning. Obtaining this intelligence is essential to gauge the level of competition and determine the strategic positioning of nano-based natural products within crowded therapeutic categories. Specifically, conventional natural products are commonly limited by poor aqueous solubility, permeability, bioavailability and efficacy. In response, respondents suggested that nano-based natural products should be strategically designed to deliver superior performance attributes, such as improved bioavailability, enhanced efficacy and safety profile, improved tolerability, faster onset of action, convenient dosing schedules, and alternative routes of administration. These enhancements are critical to facilitate market entry even in mature categories and justify the higher manufacturing and development costs typically associated with nanotechnology.

Another challenge is the potential misalignment between nanotechnology and consumer perceptions of natural products. Natural products are often perceived as affordable, safe, and minimally processed, which may conflict with the technological complexity and potentially higher pricing of nano-based formulations. Therefore, effective positioning strategies and narrative around differentiation must not only communicate the added therapeutic value but also proactively address these perceptual barriers. In particular, strategic messaging should tackle the concern that nanotechnology compromises the ‘naturalness’ of the product, and instead reframe it as a means to preserve its integrity while enhancing therapeutic effects.

Another critical concern was the lack of robust evidence to support differentiation claims. To address this, respondents advocated for the use of appropriate comparators in preclinical and clinical studies to reinforce product differentiation claims and build stakeholder confidence. Instead of comparing nanoformulations to placebo, respondents emphasized the importance of head-to-head comparisons with conventional natural products at equivalent concentrations, supported by quantitative metrics to substantiate claims of superiority. Key metrics such as bioavailability ratios, time to maximum concentration (T_max_), therapeutic index, and patient-reported outcomes were considered critical to objectively demonstrate the added value of nanoformulations. Consumers may only be willing to pay more if the nano-based formulations offer visible or measurable improvements over conventional natural products.

However, respondents cautioned that differentiating beyond the optimal trade-off between performance and cost may have adverse effects on product desirability. A key challenge identified was the misalignment between product enhancements and market affordability. While improved functionality is important, pricing must remain proportionate to the perceived benefits, especially in the current economic climate where cost sensitivity and budget constraints continue to influence healthcare spending. Product differentiation should focus on delivering meaningful and measurable improvements without overengineering features that complicate manufacturing and inflate costs. In parallel, respondents emphasized balancing innovation with simplicity, financial feasibility, and practical manufacturability, which encompasses factors such as scalability, reproducibility, process complexity, equipment availability, production yield, and quality control.

**Table 2:** Product differentiation and positioning

Key Themes	Sub-Themes	Exemplar Quotations
Product Differentiation and Positioning	Competitor landscape analysis	“When you want to launch a new product into the market, you need to know about the market demand and also who are your competitors. We will do the SWOT analysis on our competitor products, including their pricing and target audience.” (R13)
	Clear differentiation from competitors	“The researchers might have a very novel discovery in terms of formulation or product, but the same issues that the product is targeting are already being solved by a current product, and most of the researchers struggle to set a difference between their product and the current products.” (R8)
	Optimal trade-off between performance and price	“Are the risks and rewards of this new dosage form proportionate with the pricing margin that you are asking for?” (R9)
	Using appropriate comparators to substantiate differentiation	“A lot of times, I think researchers take the easy way out and they compared their products to placebo. From a marketer point of perspective, we don’t want you to compare to placebo.” (R8)

### Key theme 3: partnership and collaborative networks

The third theme of the interview highlights the importance of establishing partnerships and collaborative networks to maximise the likelihood of commercial success of nano-based natural products (Table 3). Respondents acknowledged that it is unlikely for a single firm to have the capability to develop and commercialise innovations internally, especially for highly complex innovations such as nanotechnology. They described various points along the development and regulatory pathway where nano-based natural products are especially vulnerable to failure. To overcome this, respondents underscored the importance of forming long-term strategic partnerships that span academia, industry, regulatory experts, and manufacturing specialists.

One of the most impactful collaborations mentioned by the respondents is industry-academia collaboration. Respondents recognised that academia excels in curiosity-driven fundamental research, but places less emphasis on the commercial aspects of these research. In contrast, the pharmaceutical industry involves rigorous applied research to develop useful and commercially attractive products into the market for profit. Respondents considered the industry more experienced in the entire process of drug development. Additionally, it possesses the manufacturing, marketing, and distribution capabilities needed to launch new products into the market. These resources are pivotal to ensure that scientific advances in nanotechnology are translated into real-world impact. Thus, respondents highlighted the benefits of formalizing such collaborations through structured partnership models to foster accountability, streamline decision-making, and align long-term goals. Additionally, a clear delineation of IP rights, role expectations, and conflict resolution mechanisms is vital to ensure trust and continuity across different phases of development.

Respondents also pointed to uncertainty in regulatory expectations as a barrier to commercialisation. Given the fragmented and evolving regulatory requirements for nanomaterial-based products, respondents recommended engaging regulatory experts early in the R&D process to align research activities with the requirements from authorities such as the U.S. Food and Drug Administration (FDA) and European Medicines Agency (EMA). These collaborations help interpret relevant guidelines, clarify dossier and approval requirements, and establish baseline standards that satisfy multiple regulatory authorities simultaneously. Importantly, early engagement with regulatory experts allows researchers to proactively identify and address regulatory risks that could otherwise delay market access.

Another critical issue raised by the respondents is the incompatibility between lab-scale and industrial-scale processes. The R&D phase often involves low-volume production that enables precise control over the physicochemical properties of nanoparticles. However, most lab-scale nanoformulation processes are not readily translatable to industrial-scale production, risking the loss of desired CQAs such as particle size, surface charge, shape, encapsulation efficiency, release profile, stability, and sterility. To mitigate this, respondents stressed the need for early-stage alignment with experienced nanomedicine manufacturers equipped with a comprehensive understanding of the Quality-by-Design (QbD) framework, ensuring robust, reproducible, and scalable manufacturing with strict adherence to the Good Manufacturing Practice (GMP) standards.

Another major concern was the high cost of raw materials and nano-manufacturing, which limits financial feasibility, especially for natural products often expected to be affordable. Thus, respondents suggested establishing horizontal collaboration with peer companies. This enables the optimisation of supply chain by participating in collaborative procurement with companies operating at the same level of the supply chain, thereby lowering total procurement cost through economies of scale. This is particularly relevant in nanotechnology, where the production of nanoparticles requires specialty raw materials. Beyond reducing procurement expenses, such partnerships also offer a platform for companies to share expertise and collectively evaluate pricing strategies, cost of goods, and production economics, all of which are key determinants of the commercial viability of nano-based natural products. Ultimately, these cost-reduction and value-alignment strategies contribute to a more competitive end-product price, a critical factor for consumer acceptance of nano-based natural products.

In summary, this theme highlights that a solid, multi-disciplinary, and long-term collaborative network, extending from the R&D phase to commercialisation, is beneficial as it pools complementary expertise and resources to advance innovations into commercially viable and scalable products. These collaborations are essential for de-risking the journey from laboratory to market.

**Table 3:** Partnership and collaborative networks

Key Themes	Sub-Themes	Exemplar Quotations
Partnership and Collaborative Network	Industry-academia collaboration	“Researchers are good in doing research and preparing the scientific work, but I think in terms of commercialising certain product out of research, they will still need another partner, a commercialisation partner, a company that is more adapted into the market and already has established pipeline to distribute products or sell the products” (R8)
	Collaboration with experienced manufacturers for scalable and regulatory-compliant production	“A lot of times the researchers might have a good plan, but they struggle to find a very cost-effective manufacturing partner or the quality of the manufacturing may not be as good, then their product ends up being half-hearted, and it didn’t achieve what the researchers want to achieve at the first place.” (R8)
	Strategic horizontal collaboration between peer companies	“If you purchase large quantities, you get a cheaper price correct? If you are purchasing less so it will definitely be costlier. Perhaps you can work with another company, like collaborate with them and you share some of the ingredients.” (R7)

## Discussion

**Fig. 3:**
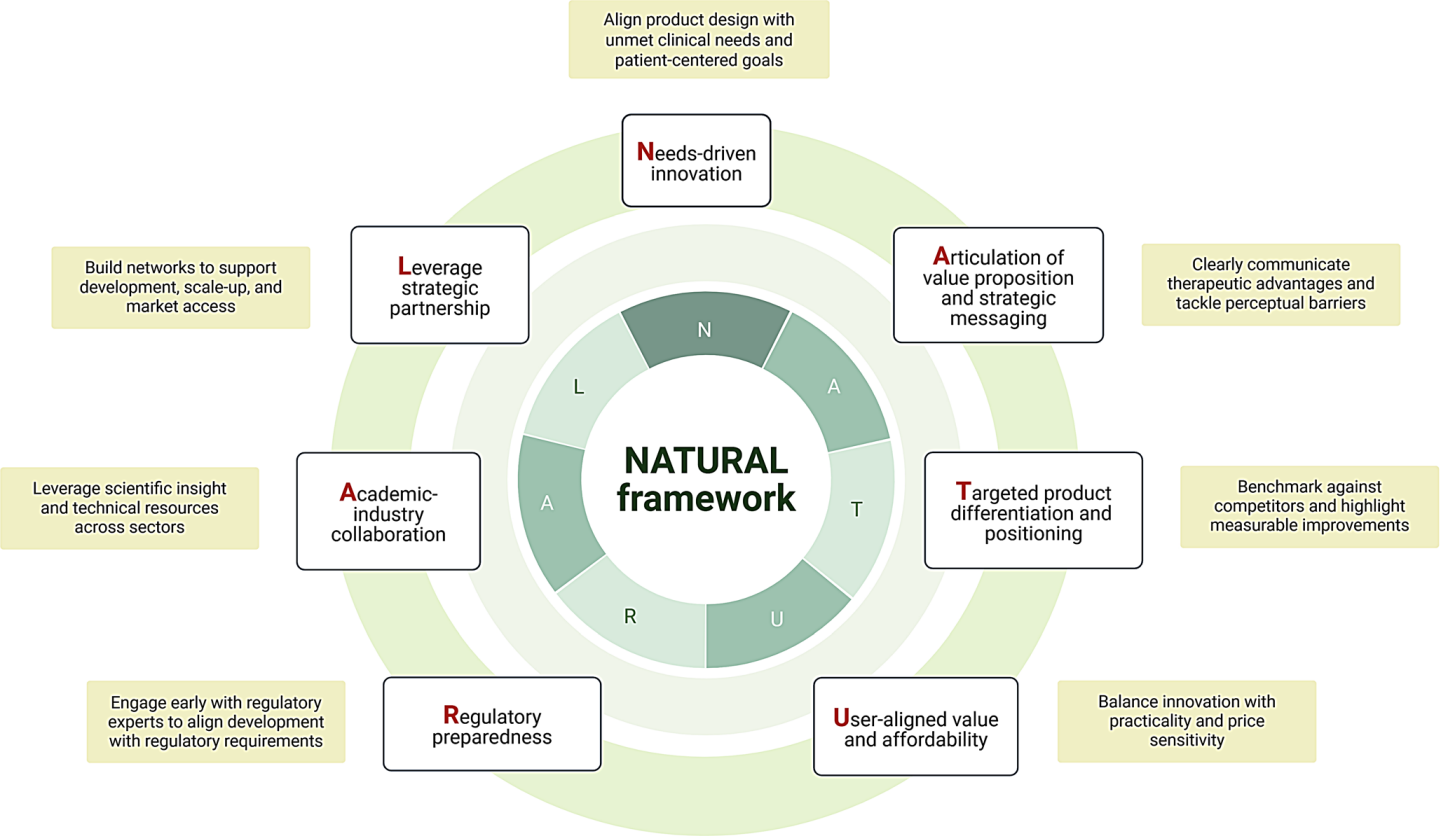
The NATURAL framework for translational planning. This figure was created with BioRender (https://biorender.com/)

Despite the surge of interest in nanomedicine since the approval of Doxil™, the translation of promising nanoformulations into marketable therapies remains limited. This persistent translational gap, often referred to as the ‘Valley of Death’, highlights the importance of understanding commercial and strategic barriers beyond technical feasibility. Notably, nearly one-quarter of investigational drug failures have been attributed to commercial and strategic issues rather than scientific limitations [10, 11]. To address this gap, this study aimed to explore the key challenges and enablers for commercialising nano-based natural products, focusing on insights from pharmaceutical stakeholders across multiple functions and sectors, including manufacturing, marketing, business development, R&D, community pharmacy, and digital health. By capturing perspectives from those directly involved in product development and market delivery, this study aimed to highlight underreported considerations that influence translational planning, strategic positioning, and commercial viability. Our thematic analysis revealed three interdependent factors that shape the commercial success of nano-based natural products: ensuring product-market fit, achieving effective product differentiation and positioning, and establishing partnership and collaborative networks. To capture the multi-dimensional strategies identified by participants, we synthesized our findings into the NATURAL framework (Fig. 3). A detailed summary of these themes, associated challenges, and strategic enablers is provided in Table 4.

**Table 4:** Summary of key themes, commercialization challenges, and proposed strategies for nano-based natural products

Key Theme	Challenge	Strategy Proposed
Product-Market-Fit	• Misalignment with actual medical needs • Limited market analysis expertise in academic teams • Disconnect between R&D efforts and commercial reality • Weak justification for premium pricing • Inability to articulate value proposition to stakeholders • Researchers unaware of investor expectations • Investor concerns over variability in natural product quality, efficacy, sourcing, and intellectual property protection	• Identify disease areas with unmet medical needs and gather real-world clinical feedback to guide innovation • Foster structured collaboration between R&D and marketing teams early in development to ensure commercial considerations inform research priorities • Clarify the clinical value added and support with pharmacoeconomic modelling • Develop value proposition with marketing input and highlight potential label claims • Develop strong business strategy and investor-aligned value propositions • Standardize sourcing and demonstrate batch consistency, robust and reproducible therapeutic benefits, and a clear intellectual property advantage of nano-based natural products
Product Differentiation and Positioning	• Insufficient product distinction from existing options • Difficulty entering crowded therapeutic market • Perception that nanotechnology compromises the “naturalness” of natural products • Insufficient evidence to support differentiation claims • Misalignment between product enhancement and market affordability • Neglecting practical considerations and pursue overly complex designs for differentiation	• Conduct competitor landscape analysis using tools like SWOT to identify areas for meaningful differentiation • Prioritize features that improve therapeutic performance to gain competitive edge and facilitate market entry • Develop strategic messaging that reframes nanotechnology as a means to preserve integrity and enhance therapeutic effects • Use appropriate comparators (e.g. conventional natural products) and quantitative metrics to support superiority claims of nano-based formulations • Strike a balance between innovation and cost-efficiency to deliver meaningful improvements while maintaining affordability • Balance innovation with simplicity, financial feasibility, and practical manufacturability
Partnership and Collaborative Network	• Limited internal capacity to develop and commercialize nano-based natural products • Disconnect between academic innovation and commercial application • Unclear regulatory expectations and fragmented global requirements • Incompatibility between lab-scale and industrial-scale nanoformulation • High cost of raw materials and nano-manufacturing processes	• Establish long-term strategic partnerships across academia, industry, regulatory experts, and manufacturers to pool resources, expertise, and infrastructure • Formalize academia-industry collaborations with clear delineation of role expectations, intellectual property rights, and conflict-resolution mechanisms • Engage regulatory experts early in the R&D process to align development with regulatory requirements and streamline dossier preparation • Partner with experienced nanomedicine manufacturers familiar with Quality-by-Design (QbD) framework and Good Manufacturing Practice (GMP) standards • Establish horizontal collaboration with peer companies to participate in collaborative procurement and share expertise to jointly evaluate pricing strategies, cost of goods, and production economics

Respondents consistently stressed the need for early and thorough market analysis to guide research planning and mitigate commercial risk. Given the growing evidence of declining R&D productivity, it is necessary to adopt a cautious approach as drug development is a lengthy and costly process [14–17]. Notably, approximately 22% of investigational drug failures in late-stage clinical development stem from commercial and strategic reasons, which is comparable to drug failures due to safety concerns [10, 11]. This is exemplified by the 5R framework adopted by AstraZeneca in 2011, which emphasised that the “Right commercial potential” is a key determinant of project success. In fact, this framework contributed to a fourfold increase in the overall project success rate between 2012 and 2016 compared to 2005 and 2010 [18, 19]. Similarly, the Guidelines On Target Assessment for Innovative Therapeutics (GOT-IT) working group recommends that academic drug discovery integrate strategic and commercial considerations from the outset [20]. These examples collectively support the need to assess marketability early in the development of nano-based natural products. However, AstraZeneca also raised concerns regarding the possible counterproductive effects of overemphasising commercial forecasting as commercial valuations may not be accurate by the time of product launch [19].

While success at every stage of the drug development is important, making the right strategic choices early is especially critical for nano-based natural products due to the intrinsic complexities of both natural product-based R&D and nanotechnology [5, 7]. Among the strategies discussed by respondents, identifying unmet medical needs was considered the most critical step in assessing commercial potential. This involves analysing disease areas which are insufficiently covered by current treatments (morbidity, mortality, disease symptoms, severity and burden), limitations of existing treatments or formulations (side effects, inconvenience) and patient perspectives [21]. This prioritisation of unmet medical needs supports the principle of needs-driven innovation, represented by the ‘**N**’ in our proposed NATURAL framework. The clinical use of natural products is commonly constrained by their poor water solubility, low permeability, and limited bioavailability [7, 8]. For instance, the anticancer alkaloids paclitaxel and docetaxel were formulated in toxic solubilising agents (Cremophor EL and polysorbate 80) to enable parenteral administration [22]. These limitations of natural products represent both unmet medical needs and commercial opportunities that nanotechnology can address.

However, successfully leveraging these opportunities requires more than scientific innovation, it demands clear articulation of value propositions and strategic messaging that resonate with stakeholders, especially investors. Respondents stressed that the anticipated benefits of nanoformulation, such as improved efficacy, faster onset, better tolerability, or more convenient dosing, must be translated into commercially compelling and persuasive narratives. These narratives help justify higher pricing, support market access, and increase the likelihood of investment and partnership. Reflecting the first ‘**A**’ in the NATURAL framework, the ability to articulate a clear value proposition through strategic messaging is fundamental to ensuring translational viability and commercial appeal.

However, respondents noted that scientific researchers often lack the commercial expertise to perform in-depth market analysis or to craft narratives that resonate with investors and stakeholders. This underscores the necessity for regular interaction between R&D and marketing teams to stay responsive to evolving market trends and capture new opportunities to innovate. Traditional marketing views markets as pre-existing regularity in which opportunities are discovered. However, newer perspectives suggest that markets are malleable and complex system consisting of a network of market actors that constantly engage and influence each other [23]. These complexities are compounded by the dynamic nature of consumers’ preferences and attitudes, which can present either opportunities or threats depending on how they are interpreted and managed [24]. As researchers may not be equipped to navigate these dynamics independently, strategic collaboration with marketing professionals becomes essential. Such collaboration is crucial to bring essential insight into evolving market dynamics, customer preferences, competitive positioning, and pricing strategies, all of which are vital for shaping compelling and investment-ready value propositions.

Another important strategy elaborated by respondents is the need for robust competitor landscape analysis to enable effective product differentiation and positioning, as reflected by the “**T**” in the NATURAL framework. In therapeutic areas where multiple companies are developing similar interventions, it is critical to evaluate whether the potential product can capture a distinct market segment, either by being the first-in-class or offering sufficient improvements on existing drugs to be considered best-in-class. A commonly used method is SWOT (strengths, weaknesses, opportunities, and threats) analysis, which helps evaluate competitors’ product attributes, market strategies, pricing, and target demographics. This enables the identification of potential differentiating features that justify commercial value, attract investors, and support sound investment decisions.

An influential analysis has shown that first-in-class drugs generally perform better commercially and capture greater market share than best-in-class drugs launched later [25]. Nevertheless, there are notable exceptions where late entrants have outperformed existing drugs by demonstrating clear and significant therapeutic advantages. One of the most prominent examples is atorvastatin (Lipitor^®^), a best-in-class statin that doubled the peak annual sales of every other statins despite entering the market nine years after the first statin. Similarly, the atypical antipsychotic aripiprazole (Abilify^®^), though sixth in its class, achieved exceptional commercial success due to its widest indications [25]. These cases underscore that meaningful and evidence-based differentiation can drive commercial success. This insight is particularly encouraging for nano-based natural products, which can be strategically positioned to offer superior therapeutic performance over conventional formulations, thereby penetrating the market and securing market share even in competitive categories. To support this positioning, respondents stressed that claims of superiority must be substantiated with robust technical data and measurable performance improvements. Crucially, such evidence should be derived from direct comparisons with conventional formulations rather than placebo controls, to convincingly demonstrate added value and therapeutic advantage in real-world contexts.

However, respondents also emphasized the importance of balancing differentiation with cost-effectiveness, affordability, and manufacturability. Overengineering may compromise affordability and reduce production efficiency by introducing unnecessary complexity, ultimately hindering adoption and market viability. The current global economic climate and lingering economic repercussions of the COVID-19 pandemic have significantly heightened cost sensitivity and altered consumer purchasing behaviour worldwide [26]. Thus, differentiation strategies should focus on value-driven enhancements that resonate with consumers, while also considering development feasibility such as process simplicity, scalability, reproducibility, yield, and quality control. This reflects the principle of User-aligned value and affordability, represented by the “**U**” in the NATURAL framework.

In addition to cost considerations, consumer perception was highlighted as a potential barrier to adoption. Some respondents noted a tension between the “natural” image of these products and the use of nanotechnology, which may raise concerns among consumers who perceive technological processing as compromising natural integrity. To overcome this, strategic messaging is needed to reframe nanotechnology as an enabler that enhances the purity, efficacy, and safety of natural products, rather than a threat to natural integrity. Encouragingly, the COVID-19 pandemic has led to a rise in pro-health attitudes among consumers, and evidence suggests that they are willing to pay more for nutraceuticals that offer clear improvements in efficacy and safety [27]. This reinforces the importance of defining and communicating the unique value propositions of nano-based natural products, particularly through comparative performance data against existing formulations.

Theme 3 highlighted the importance of establishing strong and long-term collaborative networks with diverse stakeholders to enable successful commercialisation. Natural product-based drug development and nanotechnology are highly complicated fields which require extensive resources and multidisciplinary expertise [7, 28, 29]. However, the necessary scientific and technological capabilities are often fragmented across different academic institutions and pharmaceutical companies (Atanasov et al., 2021). Thus, building collaborative networks is a strategic approach to gain a competitive edge by improving access to critical resources for product development and commercialisation, including knowledge, technology, funding, skilled personnel, patients and biospecimens, manufacturing capability, and marketing channel [29–31].

A close collaboration between academia and industry is indispensable for the successful commercialisation of laboratory-based achievements. Academia has historically been a major source of discoveries fundamental for innovations [32]. Academic researchers typically excel in curiosity-driven fundamental research that often identifies novel disease targets and therapeutic concepts. They are also more likely to pursue risky and difficult disease areas, as they are not constrained by shareholders’ expectations for profit [33]. However, academic researchers often lack sufficient financial capability, drug development and regulatory knowledge, manufacturing expertise, and commercial infrastructure, all of which are the strengths of industry [33, 34]. Thus, academia-industry collaborations are fundamentally complementary. There has been growing momentum for academia-industry collaborations to narrow the gap between basic research and commercialisation, especially in nanotechnology [35]. This model proved effective during the COVID-19 pandemic, where many vaccines were developed at unprecedented speed through public-private partnerships [36].

Nevertheless, such collaborations can be challenging due to the differences in culture, governance structures, priorities, expectations, and goals [20, 30, 37]. Academic researchers are typically motivated by intrinsic goals and pursue curiosity-driven fundamental research to generate new knowledge that may not have an immediate commercial impact [31]. In addition, academia measures the performance and impact of academic scientists by assessing their publication metrics, including the number of publications, citation counts, journal impact factor and h-index [33, 38]. In contrast, industry places strong emphasis on applied research to translate basic research into useful and commercially attractive products. The product- and profit-oriented management of industry remains a relatively unfamiliar concept in academic institutions and could be a source of tensions between the academia and industry [31]. These differences underscore the importance of clearly defined roles, mutual understanding, and structured agreements, particularly around IP rights and milestone responsibilities, to prevent misalignment and ensure productive collaboration. With sympathetic management and a shared vision to address unmet medical needs, academia and industry can bridge their divides to bring nano-based natural products to market, highlighting the importance of the second ‘**A**’ in our NATURAL framework.

Regulatory uncertainty emerged as a key barrier to the commercialisation of nano-based natural products, particularly given the fragmented and evolving global regulatory landscape on nanomedicines. The absence of harmonized standards across regulatory authorities such as the FDA and EMA creates complexity in regulatory dossier preparation and approval pathways [39, 40]. For instance, the lipid components in lipid nanoparticles have been variably classified as excipients, novel excipients, or starting materials by different companies. These discrepancies were accepted by the FDA but required reclassification by the EMA, exemplifying the regulatory divergence that can inflate development costs and prolong timelines [40]. These challenges may be compounded for nano-based natural products due to their hybrid nature, which can place them in overlapping regulatory domains that are potentially governed by distinct and incompatible frameworks, further complicating regulatory classification and approval processes. To mitigate these challenges, early and continuous engagement with regulatory experts is vital to de-risk development by ensuring research activities generate data that align with regulatory expectations. Notably, the European STARS (Strengthening Training of Academia in Regulatory Science) project was initiated to improve regulatory knowledge among academic researchers, while also facilitating bidirectional knowledge exchange between academics and regulators [41]. Bridging these regulatory gaps is essential to enhance the translational viability of academic innovations in nano-based natural products, aligning with the “**R**” (regulatory preparedness) in our proposed NATURAL framework.

Another key form of collaboration identified by the respondents is early-stage alignment with experienced nanomedicine manufacturers. This is important to ensure that small-volume laboratory production is upscaled successfully to large-scale industrial manufacturing while maintaining the CQAs of nanoparticles. CQAs are physical, chemical, biological, and microbiological properties of nanoparticles that must be maintained within specified limits to ensure product quality, safety and efficacy. The typical CQAs for nanomedicines include particle size and size distribution, shape and morphology, surface charge, drug loading, release kinetics, stability, sterility, and biocompatibility [39, 42]. Failure in scaling-up risks the loss of CQAs, which may lead to altered nano-bio interactions that compromise therapeutic efficacy and safety profiles of nanomedicine [43]. To overcome these challenges, early alignment with nanomedicine manufacturers experienced in QbD framework is important for early definition of CQAs, which serve as the reference framework to validate the performance and quality of the final products. These predefined CQAs help quantify and control the impact of manufacturing modifications, thereby reducing translational risk and supporting long-term success [39].

Lastly, to address the financial constraints posed by specialty raw material and complex nano-manufacturing, respondents highlighted the value of horizontal collaboration with peer companies. In contrast to vertical collaboration which involves upstream or downstream collaboration with suppliers or consumers, horizontal collaboration is the strategic collaboration with companies or competitors (coopetition) on the same level of market to perform various operations with greater success [44]. Through the formation of procurement intermediaries and collective demand pooling, horizontally aligned firms can leverage economies of scale to negotiate lower unit prices and reduce overall cost of goods [45, 46]. Furthermore, supply chain collaboration enhances supply chain resilience against unexpected supply chain disruptions in specialty materials, which ensures responsiveness and competitiveness in an increasingly turbulent environment [47–49]. Notably, evidence from video game industry demonstrates that horizontal collaborations enables information sharing and knowledge exchange, which significantly improve the market performance of both incremental and radical innovations [50]. This finding is relevant to our study, as horizontal collaboration between companies with complementary expertise, such as between nanotechnology firms or between natural product companies, may enable resource pooling, knowledge sharing, and joint problem-solving, all of which are critical for the successful development and differentiation of innovative nano-based therapeutics. These forms of collaboration exemplify leveraged strategic partnerships, as represented by the “**L**” in our proposed NATURAL framework. Nevertheless, achieving the desired results remains challenging due to the risk of knowledge leakage, power imbalances, and conflicting objectives [47, 51]. Successful horizontal collaboration in complex and IP-sensitive field, such as nano-based natural products, requires firms to adopt mechanisms that balance knowledge sharing and protection, such as organizational designs integrated with knowledge protection mechanisms and active knowledge flow management [52, 53].

### Limitations and future perspectives

While this study provides insights into the challenges and enablers of clinical translation of nano-based natural products, several limitations warrant considerations. Firstly, as a qualitative study based on interviews, the findings reflect the specific perspectives and experiences of selected participants, which may limit their generalisability. Although the sample included diverse professional backgrounds, the absence of other key stakeholders, such as regulatory authorities, clinicians, consumers, and investors, may limit the comprehensiveness of the findings. These groups represent critical stakeholders in the commercialization landscape and are likely to offer distinct perspectives on regulatory hurdles, clinical utility, and consumer acceptability. In particular, integrating consumer insights through focus groups, surveys, or willingness-to-pay studies may help assess market acceptance and price sensitivity, which are vital to evaluating product-market fit. Additionally, future research would benefit from including interdisciplinary experts, such as health economists and supply chain specialists, whose insights are important for addressing systemic commercialization challenges beyond scientific and technical development.

Secondly, the study was cross-sectional and captured stakeholder insights at a single point in time. However, commercialisation landscapes and stakeholder perspectives are dynamic and may evolve alongside regulatory reforms, technological advances, and shifting market demands. Future research could benefit from longitudinal study designs to monitor how stakeholder perceptions and strategic priorities shift over time, particularly as the nanotechnology field matures and more nano-based products enter the market. Incorporating case studies of both successful and unsuccessful commercialization efforts could also help uncover critical success factors and common pitfalls. While this study qualitatively explored stakeholder insights on therapeutic value and affordability, future work could complement these findings with quantitative assessments, such as formal value evaluations, pricing strategies, and comparative economic modeling, to better predict and quantify trade-offs in nano-based natural product development.

Finally, the research was geographically limited, and future studies could benefit from broader international sampling to account for varying regulatory environments, market maturity, attitudes towards natural products and nanotechnology. Such diversity would allow for a more nuanced understanding of region-specific commercialization challenges and enablers, particularly across countries of different healthcare system, reimbursement model, and income levels, where regulatory frameworks, affordability, and innovation incentives may differ substantially.

Beyond study-specific limitations, the field of nano-based natural products faces persistent challenges that warrant broader attention. Due to the complexity and diversity of nanomedicine design, there is no universal route to successful translation, and strategies must be adapted to specific formulations, therapeutic areas, and market contexts. Future progress will require more predictive preclinical models, standardized testing protocols, and clearer regulatory guidance for nano-based natural products. Within this evolving landscape, the NATURAL framework proposed in this study should continue to evolve and be refined to accommodate emerging technologies, regulatory shifts, and market dynamics. While translational studies are critical, fundamental research remains essential to advance formulation design, mechanism elucidation, and disease targeting, thereby ensuring a robust pipeline of clinically relevant nano-based natural products.

## Conclusion

This study highlights the persistent challenges in translating nano-based natural products into clinically and commercially viable therapies. While nanotechnology offers powerful tools to overcome pharmacokinetic limitations of natural compounds, successful translation to market remains hindered by strategic, regulatory, and organisational barriers. By engaging pharmaceutical stakeholders, this study uncovered three critical enablers of success: ensuring product-market fit, achieving effective product differentiation and positioning, and establishing partnership and collaborative networks.

These findings support the growing consensus that scientific excellence alone is not sufficient. Commercialisation requires early integration of market analysis and need-driven innovation to ensure product development aligns with real-world needs and delivers compelling value propositions to both investors and end-users. Nano-based natural products must demonstrate measurable advantages compared to conventional formulations, while maintaining simplicity and avoiding overengineering to ensure affordability. Strategic use of comparators and quantitative metrics can substantiate these claims and build stakeholder confidence. Beyond demonstrating technical performance, value communication must also address consumer perception, particularly the tension between ‘natural’ identities and the use of advanced technologies like nanotechnology. Framing nanotechnology as a means to enhance the efficacy and safety of natural products is critical to market acceptance. Lastly, successful translation of nano-based natural products requires navigating strategic, regulatory, and manufacturing challenges. Our findings highlight the critical role of both cross-sector and horizontal collaborations, spanning academia, industry, regulatory experts, manufacturers, and peer companies, to pool complementary expertise and resources required to bridge scientific innovation with market readiness.

To support strategic planning in this complex landscape, we propose the NATURAL framework, which is a translational planning tool derived from the insights of pharmaceutical stakeholders in this study. The framework integrates the key enablers identified across the three themes, namely **N**eeds-driven innovation, **A**rticulation of value proposition and strategic messaging, **T**argeted product differentiation and positioning, **U**ser-aligned value and affordability, **R**egulatory preparedness, **A**cademic-industry collaboration, and **L**everage strategic partnerships. By addressing the critical gaps that often lead to failure, the NATURAL framework aims to help innovators navigate the Valley of Death by anticipating barriers, engaging relevant partners, and improving the likelihood of success. Future work may refine and validate this framework across diverse product categories, but it represents a crucial step toward bridging scientific promise with real-world impact for nano-based natural products.

## Data Availability

The datasets generated during and/or analysed during the current study are available from the corresponding author on reasonable request.
